# Mercury Removal From Aqueous Solutions With Chitosan-Coated Magnetite Nanoparticles Optimized Using the Box-Behnken Design

**DOI:** 10.17795/jjnpp-15913

**Published:** 2014-04-13

**Authors:** Nadereh Rahbar, Alireza Jahangiri, Shahin Boumi, Mohammad Javad Khodayar

**Affiliations:** 1 Nanotechnology Research Center, Jundishapur University of Medical Sciences, Ahvaz, IR Iran; 2 Department of Medicinal Chemistry, School of Pharmacy, Jundishapur University of Medical Sciences, Ahvaz, IR Iran; 3 Department of Pharmacology and Toxicology, Toxicology Research Center, School of Pharmacy, Ahvaz Jundishapur University of Medical Sciences, Ahvaz, IR Iran

**Keywords:** Chitosan, Magnetite Nanoparticles, Mercury

## Abstract

**Background:**

Nowadays, removal of heavy metals from the environment is an important problem due to their toxicity.

**Objectives:**

In this study, a modified method was used to synthesize chitosan-coated magnetite nanoparticles (CCMN) to be used as a low cost and nontoxic adsorbent. CCMN was then employed to remove Hg^2+^ from water solutions.

**Materials and Methods:**

To remove the highest percentage of mercury ions, the Box-Behnken model of response surface methodology (RSM) was applied to simultaneously optimize all parameters affecting the adsorption process. Studied parameters of the process were pH (5-8), initial metal concentration (2-8 mg/L), and the amount of damped adsorbent (0.25-0.75 g). A second-order mathematical model was developed using regression analysis of experimental data obtained from 15 batch runs.

**Results:**

The optimal conditions predicted by the model were pH = 5, initial concentration of mercury ions = 6.2 mg/L, and the amount of damped adsorbent = 0.67 g. Confirmatory testing was performed and the maximum percentage of Hg^2+^ removed was found to be 99.91%. Kinetic studies of the adsorption process specified the efficiency of the pseudo second-order kinetic model. The adsorption isotherm was well-fitted to both the Langmuir and Freundlich models.

**Conclusions:**

CCMN as an excellent adsorbent could remove the mercury ions from water solutions at low and moderate concentrations, which is the usual amount found in environment.

## 1. Background

Water, air, and soil pollution by heavy metals often originate from industry and are serious environmental problems. There is much current focus on methods of removal from natural waters and industrial waste waters to produce high quality water or to enable water recycling. Mercury is a heavy metal of primary concern because of its toxicity, persistence in the environment, and bioaccumulation. Techniques for mercury removal include traditional precipitation and coagulation methods, ion-exchange, solvent extraction, ultra filtration, and adsorption. The latter has attracted attention because of its effectiveness and ease of handling the adsorbent (1-4). Adsorbents used for mercury removal include activated carbon (5), modified chitosan (6-8), modified silica (2, 4), modified resin (9), and modified clay (10). Nanotechnology offers new and efficient ways for removal of organic and inorganic pollutants, especially in water, because of the high surface/volume ratio of nanomaterials (11-13). Among these, iron-based nanomaterials as solid phase extractors were promising in the removal of pollutants, because they are easily removed from a water solution using an external magnetic field (3, 14, 15).

Chitosan and its derivatives are effective and low-cost sorbents of heavy metals (16). Chitosan is a natural and harmless polysaccharide prepared by de-acetylating chitin widely used in food and pharmaceutical preparations and medical processes. It is capable of adsorbing a number of metals via its amino groups serving as ion-exchanges, or chelating sites, and can be easily modified by chemical and physical processes (17-23). Nanoparticles that incorporate the positive aspects of chitosan and magnetite nanoparticles (MNs) can be an effective sorbent for heavy metal removal from aqueous solutions, because there is an effective avenue to isolate nanomaterials after use (24-29).

Response surface methodology (RSM) with a Box-Behnken design (BBD) is a common statistical tool for optimization of variables affecting the removal process because of relatively small number of systematic tests required, which reduces time, cost and resources. This experimental design can assess interaction effects between factors affecting adsorption and improve the removal of analyte (30-36).

## 2. Objectives

This study developed a procedure to remove mercury ions from an aqueous solution. RSM-BBD was used to optimize the operating factors for maximum mercury removal using these nanoparticles.

## 3. Materials and Methods

### 3.1. Reagents and Solutions

Chemicals of analytical grade and double distilled water were used in this study. A stock solution of Hg^2+^ (1000 mg/L) was purchased from Merck (Darmstadt, Germany). Diluted mercury solutions were prepared using successive dilutions of the stock solution. FeCl_2_.4H_2_O, FeCl_3_.6H_2_O, ammonia solution (25%), glacial acetic acid, sodium dodecyl sulfonate, dithizone, hydrochloric acid, and sodium hydroxide were purchased from Merck (Darmstadt, Germany). Chitosan (600-1200 cp and 96% degree of deacetylation) was purchased from Primex (Iceland).

### 3.2. Preparation of Chitosan-coated Magnetite Nanoparticles

The method of preparing CCMNs was developed by modifying published procedures (37, 38). Chitosan solution (1% w/v) was prepared by dissolving 0.1 g of chitosan flakes in 0.5 mL glacial acetic acid and diluting it with 10 mL distilled water. A 5 mL mixture of ferrous and ferric chlorides with a molar ratio of 1:2 was prepared and 50 mL of 1 M ammonium hydroxide solution was added drop-by-drop while stirring vigorously. Meanwhile, the chitosan solution was slowly dripped into the mixture. The mixture was stirred for 10 minutes, the prepared nanoparticles were collected using an external magnetic field and thoroughly washed with distilled water to remove excess ammonium hydroxide.

### 3.3. Characterization Methods

The Fourier transform infrared spectroscopy (FT-IR) spectra of the MNs and CCMNs were recorded using a Perkin-Elmer spectrometer (model BX2, USA), in the scanning range of 400-4000/cm. Scanning electron microscopy (SEM) was used to show the dimensions and morphology of the nanoparticles (TESCAN, model VEGA (II) LMH, Czech Republic). Energy dispersive x-ray diffraction (EDX) patterns of the nanoparticles were obtained at room temperature on a CTS cursor (Czech Republic).

### 3.4. Mercury Removal

The uptake procedure was performed in batch mode. The adsorption experiments were performed by mixing appropriate amounts of damp nanoparticles with 50 mL of mercury solution at a given concentration. Adjustment of the solution initial pH to the desired value was performed using HCl 0.1 mol/L and NaOH 0.1 mol/L solutions. After stirring for 10 minutes at room temperature at 150 rpm, the solid phase was removed from the solution using an external magnetic field. All experiments were performed in triplicate and the results were indicated as averages. The percentage of Hg^2+^ removed was calculated as:

1) Removal, % = 100 × (C_0_ − C_e_)/C_0_

Where C_0_ and C_e_ are the initial and equilibrium concentrations of Hg^2+^ in a solution of mg/L, respectively. The metal-loading capacity of the CMN at equilibrium was determined as:

2) q_e_, mg/g = (C_0_ - C_e_) × V/W

Where V is the volume of solution (L), and W is the amount of adsorbent (g).

### 3.5. Analytical Measurements

The concentration of mercury in solution after removal was analyzed using a UV-Vis spectrophotometer (Jasco 7800, Japan). Mercury-dithizone complex formed in an acidic solution with a maximum absorbance of 490 nm. Mercury determination was performed according to the standard methods slightly modified to achieve more sensitivity (39, 40).

### 3.6. Experimental Design

A three-level, three-factor Box-Behnken experimental design was employed to verify the performance of pH (5-8), the initial concentration of Hg^2+^ (2-8 mg/L), and the amount of damp adsorbent (0.25-0.75 g) in mercury removal and to determine optimum levels of these parameters. The Experimental range and level of independent variables are shown in Table 1. The percentage of Hg^2+^ removed was taken as the response of the system. Minitab 15 software was used to design the experiments. Table 2 shows the experimental design derived from BBD and the results of all 15 experiments, including three center points. Each experiment was performed in triplicate to verify reproducibility. The results were used to calculate the 10 coefficients of the second-order polynomial equation. This equation shows the relation between the desired response and the independent variables (pH, initial mercury concentration, and amount of adsorbent). Considering all linear, square, and linear-by-linear interaction terms, the second-order polynomial equation can be described as:

3) Y = b_0_ + b_1_x_1_ + b_2_x_2_ + b_3_x_3_ + b_12_x_1_x_2_ + b_13_x_1_x_3_ + b_23_x_2_x_3_ + b_11_x_1_^2^ + b_22_x_2_^2^ + b_33_x_3_^2^

where Y is the response (percentage of Hg^2+^ adsorbed); b0 is the offset term; b_1_, b2, and b_3_ are the linear coefficients; b_11_, b_22_, and b_33_ are the quadratic coefficients, and b_12_, b_13_ and b_23_ are the coefficients of the linear-by-linear interaction effect between independent variables x_1_ (pH), x_2_ (initial concentration of Hg^2+^ solution),and x_3_ ( amount of adsorbent) (41). The goodness of fit of the model was assessed using a coefficient of regression (R^2^) and analysis of variance (ANOVA).

**Table 1.** Experimental Range and Level of Independent Variables

Factors Range and Levels (Coded)	-1	0	1
**Hg^+2^ concentration,mg/L**	2	5	8
**pH**	5	6.5	8
**Wet absorbent amount, g**	0.25	0.50	0.75

**Table 2.** Box-Behnken Design Matrix for Three Variables-three Levels Together With Observed and Predicted Values

Exp. Run	Hg^+2^ conc., mg/L	pH	Adsorbent Amount	Removal, %	Predicted
**1**	8	6.5	0.25	95.0	94.625
**2**	2	6.5	0.25	90.7	91.025
**3**	5	5.0	0.25	96.8	97.200
**4**	2	5.0	0.50	97.4	96.675
**5**	5	6.5	0.50	97.8	97.800
**6**	8	5.0	0.50	98.9	98.875
**7**	5	8.0	0.25	95.1	94.750
**8**	2	8.0	0.50	88.7	88.725
**9**	5	8.0	0.75	95.6	95.200
**10**	2	6.5	0.75	89.6	89.975
**11**	8	6.5	0.75	99.5	99.175
**12**	8	8.0	0.50	98.6	99.325
**13**	5	6.5	0.50	97.8	97.800
**14**	5	5.0	0.75	99.9	100.250
**15**	5	6.5	0.50	97.8	97.800

## 4. Results

### 4.1. FT-IR and SEM Analysis for Adsorbent

**Figure 1.**
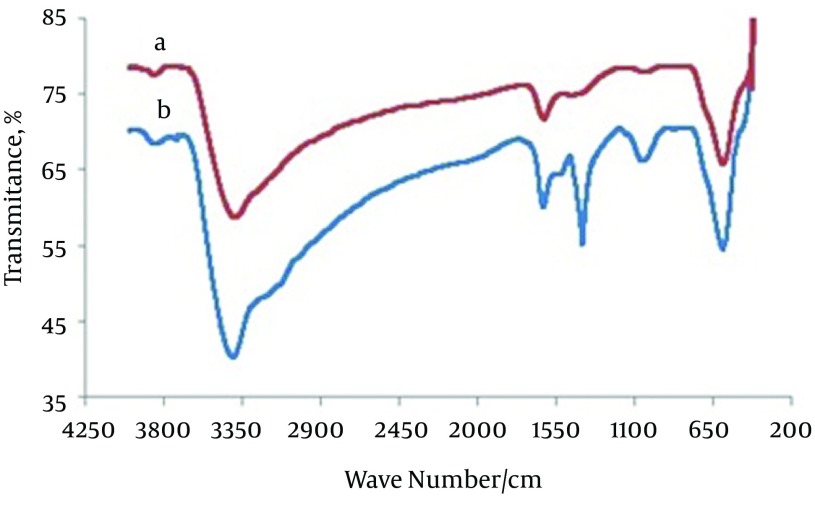
FT-IR Spectra of: A) Fe_3_O_4_ Nanoparticles, B) CCMN

**Figure 2.**
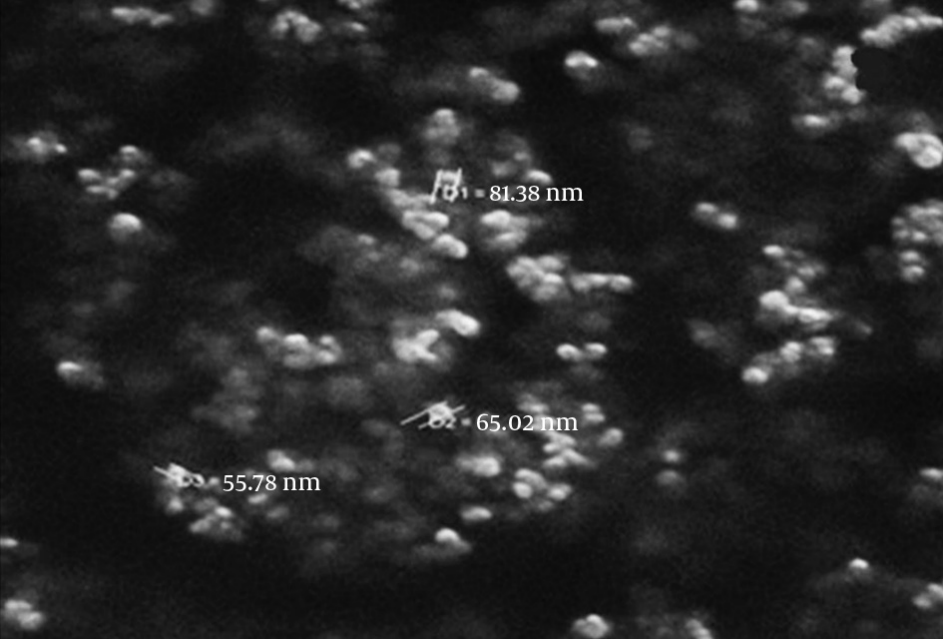
The SEM Photograph of CCMN

Figure 1 shows the FT-IR spectra for (a) magnetite nanoparticles and (b) CCMN. The major bands in Figure 1A at 3392 and 598/cm are caused by the O-H stretching vibrations of the adsorbed water on the magnetite nanoparticles and the characteristic peak of Fe_3_O_4_, respectively. Figure 1B shows the adsorption band at 3406/cm caused by N-H and O-H stretching vibrations. The peaks at 1627 and 1403/cm correspond to the N-H bending vibration and C-N stretching vibration, respectively (25, 26, 37). The SEM micrographs for the magnetite nanoparticles and CCMNs are shown in Figure 2. As shown in Figure 2A, Fe_3_O_4_ particles have a spherical shape with a diameter distribution of 30-100 nm. Figure 2B indicates that the CCMN sizes are slightly larger than those of the magnetite particles. This observation is a result of chitosan coating the magnetite nanoparticles. The size of the particles remained in the nanometer range after coating, thus achieving the goal of preparing chitosan-coated nanoparticles.

### 4.2. Box-Behnken Statistical Analysis

ANOVA and α level of 0.05 (95% confidence) were used to determine the statistical significance of the independent variables and their interactions. The second-order polynomial coefficients and statistical parameters were analyzed using Minitab 15 software to describe the results. ANOVA for the quadratic model for mercury adsorption onto the CCMNs is shown in Table 3. The regression model F value of 46.63 and α value ˂ 0.001 are highly significant. ANOVA showed that all effects were statistically significant (P < 0.05) at 95% confidence levels, except for the first order main effect of the initial concentration of mercury ions (P = 0.889), the second-order pH (P = 0.124), and the interaction effect of pH and the amount of adsorbent (P = 0.102). The predicted R^2^ of 0.9880 and adjusted R2 of 0.9670 were in reasonable agreement, indicating the significance of the model (30). The regression coefficient for the model (0.9882) showed the goodness of fit of the model and that only 1.2% of the variation could not be explained by the regression model. Moreover, the high value of predicted regression coefficient (0.9880) showed a good correlation between experimental results and predicted responses. Using multiple regression analysis, an empirical association was observed between the percentage of mercury ions removed as the response (Y) and the three experimental variables as shown in below equation:

4) Y = 111.464-0.094 C-6.328 pH + 30.633 W + 0.467 pH × C-1.733 pH×W 1.867 C × W + 0.278 pH^2^-0.281C^2^-25.200 W^2^

Where C is the initial concentration and W is the amount of adsorbent. The final mathematical model of significant actual factors for Hg^2+^ removal (Y) by the CCMNs determined by Minitab software is (30):

5) Y = 111.464-6.328 pH + 30.633 W + 0.467 pH × C + 1.867C × W-0.281C^2^-25.200 W^2^

From equation 5, it can be concluded that the first order main effects of pH and amount of adsorbent had significant effects on removal, while the same effect for the initial concentration was insignificant (P ˃ 0.05). ANOVA results (Table 3) showed that first-order effects of the main factors were more significant than their quadratic and interaction effects. Moreover, of all model components, the second-order initial concentration showed the lowest effect on Hg^2+^ removal efficiency (P = 0.889).

**Table 3.** ANOVA for Response Surface Reduced Quadratic Model

Term	Coefficient	SE Coefficient	T	P Value
**Constant**	111.464	7.61163	14.644	< 0.001
**pH**	-6.328	2.03878	-3.104	0.027
**W**	30.633	8.15404	3.757	0.013
**C**	-0.094	0.64340	-0.147	0.889
**pH × pH**	0.278	0.15025	1.849	0.124
**W × W**	-25.200	5.40913	-4.659	0.006
**C × C**	-0.281	0.03756	-7.469	0.001
**pH × W**	-1.733	0.86615	-2.001	0.102
**pH × C**	0.467	0.07218	6.465	0.001
**W × C**	1.867	0.43308	4.310	0.008

### 4.3. Effect of Parameters on Mercury Removal

The most important factors affecting mercury ion adsorption onto CCMN were pH, initial concentration of mercury ions, and amount of adsorbent. Surface and contour plots of these parameters showed their association with Y. In these plots, the function of two factors is examined, while the third factor is held at a constant level.

#### 4.3.1. pH Effect

**Figure 3.**
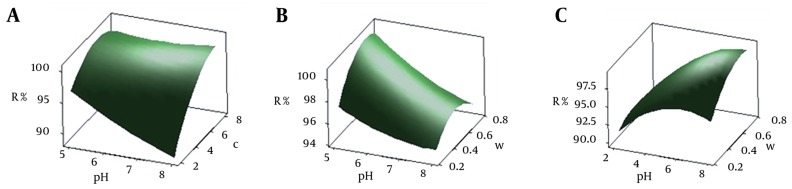
Three-dimensional Response Surface Plot A) Combined effect of pH and initial concentration; B) Combined effect of pH and adsorbent amount and; C) Combined effect of initial concentration and adsorbent amount

The pH values ranged from five to eight. Based on ANOVA analysis, initial pH had the greatest negative effect on adsorption. Increasing pH decreased the uptake of mercury ions. Figure 3A and 3B represent the interactive effects of pH by initial concentration of mercury ions and amount of adsorbent, respectively, on the percentage mercury ions removed as analyzed by BBD. Figure 3A shows that, as pH increased from five to eight, with metal concentration and CCMN levels kept constant, the percentage of adsorption decreased. BBD model predicted that the highest uptake of mercury should be at pH = 5 as the optimum value. This result agrees with those of the previous studies (6, 7, 27). The results of previous studies and those obtained from this study indicate that a mixture of two mechanisms might be responsible for the uptake of mercury ions by CCMN. The presence of amino groups in chitosan (pK_a_ = 6.5) helps it to adsorb transition metals via ion exchange (low pH) and complex formation mechanisms (high pH) (42). From the chitosan pK_a_ value, it can be assumed that, where pH = 5, amino groups on the surface of the adsorbent are protonated and adsorb mercury ions via complex formation. It has also been suggested that, for low pH in the presence of HCl, the abundance of H^+^ leads to formation of anion complexes, such as HgCl_3_^¯^. This anion can be adsorbed onto the CCMN via an ion exchange mechanism (7, 27). For higher pH values, the retention of Hg^2+^ decreased, probably because of the formation of metal hydroxide species (7).

#### 4.3.2. Initial Mercury Concentration

Mercury adsorption onto CCMN was tested at initial concentrations ranging from 2 to 8 mg/L. Figure 3A and 3C show the combined effects of initial mercury concentration with pH and amount of adsorbent. The removal percentage increased with increasing the initial mercury concentration and increased the percentage of mercury ions removed for values from to 2 to 6 mg/L and then decreased slightly for higher values. This finding is in agreement with previous studies and can be attributed to the driving force that overcomes all mass transfer resistance of metal between the aqueous and solid phases (29, 33, 43, 44). Furthermore, it can be assumed that increasing initial metal concentration increases the number of collisions between mercury ions and CCMNs, thus increasing adsorption. BBD analysis predicted that the maximum removal of mercury ions would occur for an initial mercury concentration of 6.2 mg/L.

#### 4.3.3. Adsorbent

The results of the combined effects of the amount of adsorbent with pH and initial metal concentration are shown in Figure 3B-D response surface plots and contours. Figure 3B shows that increasing the amount of adsorbent from 0.25 g to 0.75 g while keeping the pH = 5 and initial mercury concentration constant (6.2 mg/L), increases the percentage of removal of Hg^2+^ by CCMN. The higher mass of adsorbent means that more surface area, including functional groups, is available. BBD analysis predicted the optimum amount of damp adsorbent to be 0.67 g, which is equivalent to 67 mg of dried CCMN. Finally the optimum values for independent variables of pH, mercury and amount of adsorbent were 5, 6.2 mg/L and 67 mg, respectively.

### 4.4. Kinetic Studies

The effect of contact time on the adsorption of mercury ions under optimal conditions (pH = 5, initial metal concentration = 6.2 mg/L and amount of damp adsorbent = 0.67 g) was studied. The results showed that the adsorption rate was high and reached equilibrium in < 5 minutes with 99.91% of the mercury ions adsorbed. This rapid adsorption might be due to chemical binding or electrostatic attraction between mercury ions and surface functional groups (4). Three kinetic models (pseudo first-order, pseudo second-order, and intra-particle diffusion) were used to test the experimental data and verify the kinetic mechanism of sorption. The linear form of the pseudo second-order equation is:

6) t/q_t_ = 1/k_2_q_e_^2^ + t/q_e_

**Figure 4.**
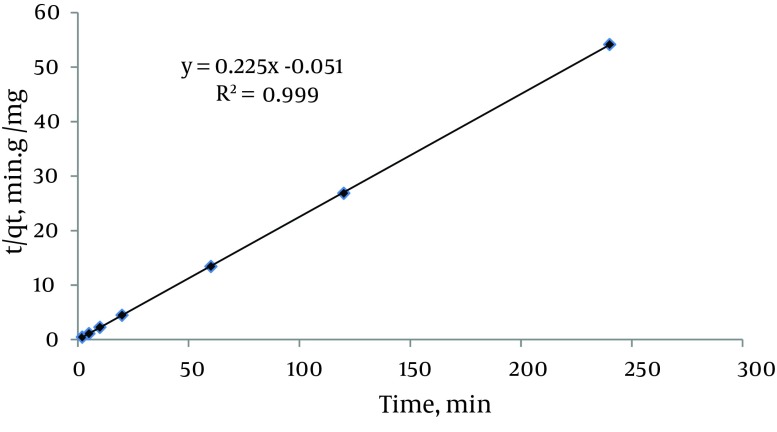
Pseudo Second Order Kinetic Plot for the Adsorption of Hg^2+^ on CCMN (Initial Concentration 6.2 mg/L, CCMN Amount 67 mg and pH = 5)

Where k_2_ (g/mg/min) is the pseudo second-order rate constant of adsorption. A plot of t/q_t_ versus t (Figure 4) yielded a very good straight line and correlation coefficient. In addition, q_e_ (4.44 mg/g) agreed well with the calculated q_e_ (4.48 mg/g) value revealing that adsorption of Hg^2+^ onto the CCMN follows a pseudo second-order mechanism. This trend suggests that a chemisorption reaction via the amino groups on the surface of the CCMN, a high specific surface area, and the absence of internal diffusion predominate in the rate controlling step. It is likely that sharing of electrons between anions and the adsorbent produced valence forces in the adsorption process (26, 27). These results are consistent with other investigations (37).

### 4.5. Adsorption Isotherms

It is important to investigate equilibrium adsorption isotherms in the design of adsorption system and to explain the interactive behavior of the metal ions and solid phase. To generate equilibrium adsorption data in this study, the Langmuir and Freundlich isotherm models were employed. To study these adsorption isotherms, initial mercury ion concentrations were set in the range of 1-15 mg/L under optimal conditions (pH = 5, and amount of wet adsorbent = 0.67 g). The Langmuir isotherm model is expressed as:

7) C_e_/q_e_ = C_e_/q_m_ + 1/K_L_q_m_

Where C_e_ (mg/L) is the equilibrium concentration of the metal ions, q_e_ (mg/g) is the percentage of metal ions adsorbed under equilibrium conditions, q_m_ (mg/g) is the maximum adsorption capacity, and K_L_ (L/mg) is the Langmuir constant (a measure of the affinity of binding sites and is a measure of the adsorption energy). The Langmuir isotherm assumes that uptake occurs on a homogeneous surface by monolayer adsorption without interaction between the absorbed materials. Plotting C_e_/q_e_ against C_e_ gives a straight line and q_m_ and K_L_ can be calculated from the slope and intercept of the plot, respectively. Another important parameter, the separation factor (R_L_), shows a degree of suitability of the adsorbent toward the metal ions and is calculated using binding constant K_L_ as:

8) R_L_ = 1/(1 + C_i_K_L_)

Where C_i_ is the initial concentration of the metal ion. The adsorption process can be defined by the magnitude of R_L_ as: R_L_ > 1.0 is unsuitable; R_L_ = 1 is linear; 0 < R_L_< 1 is suitable; R_L_ = 0 is irreversible (27). The Freundlich isotherm differs from the Langmuir isotherm model. It describes multilayer adsorption and adsorption on heterogeneous surfaces. The experimental data was fitted to the linear Freundlich equation as:

9) logq_e_ = log K_F_ + 1/n log C_e_

Where K_F_ (mg/g) and 1/n (L/mg) are the Freundlich constants for adsorption capacity and energy of adsorption, respectively. Table 4 presents the parameters obtained by implementing the Langmuir and Freundlich models using the experimental data. As shown in Table 4, correlation coefficient R^2^ for the Langmuir isotherm is 0.949, which indicates that the adsorption of mercury onto the CCMN is favorable. The R_L_ calculated for 1-15 mg/L concentrations of mercury ions at pH = 5 and damped adsorbent = 0.67 g lie between 0.01 and 0.13, indicating that Hg^2+^ adsorption onto CCMN was linear.

**Table 4.** The Langmuir and Freundlich Parameters for Adsorption of Hg^2+^ Onto CCMN

	Langmuir	Freundlich
**q_max_, mg/g**	9.34	-
**K, L/mg**	6.69	7.01
**R^2^**	0.949	0.934
**R**	0.01-0.13	-
**1/n**	-	0.41

Table 4 shows that the plot of the Freundlich isotherm had an acceptable fit with a correlation coefficient of 0.943, suggesting the presence of heterogamous conditions. The small value of 1/n (0.407), between 0 and 1, and the large value of KF (7.01 mg/g) show that mercury ions can be effectively adsorbed by CCMN (4, 45, 46). It can be concluded that both isotherm models are adequate to describe the adsorption process mechanism for the studied concentration range.

## 5. Discussion

In this study, the Box-Behnken methodology was used to find the feasibility and the adsorbent for the removal of mercury ion from aqueous solutions. This approach proved to be an effective and time saving method to study the influence of major process factors (pH, concentration, amount of adsorbent) and determine optimal conditions for the removal of mercury ions while significantly reducing the required number of experiments. This model indicated that 99.91% removal of Hg^2+^ is possible at optimal conditions of pH = 5, mercury ion concentration = 6.2 mg/L, and amount of damped adsorbent = 0.67 g (equivalent to 67 mg of dried CCMN). The results showed that CCMN is an excellent adsorbent for low and moderate concentrations of mercury ions, which is the usual amount found in water and waste aqueous solutions.

The adsorption isotherm was well fitted by both the Langmuir and Freundlich models. Kinetic studies of the adsorption process confirmed the efficiency of the pseudo second-order kinetic model. The high initial adsorption rate and short equilibrium uptake time indicated that the surface of CCMN has a high density of active sites for mercury ion uptake. This proposed method applies an environmentally friendly non-toxic adsorbent which does not threaten human health. In addition, the critical step of separation of the treated solution from the adsorbent can be accomplished easily using an external magnetic field.
